# A New Cyanobacterium, *Pseudoaliinostoc murmanicum* (Nodulariaceae), from the Russian Arctic Technogenic Habitats

**DOI:** 10.3390/plants15081166

**Published:** 2026-04-09

**Authors:** Denis Davydov, Anna Vilnet

**Affiliations:** Polar-Alpine Botanic Garden-Institute—Separate Subdivision of Federal Research Centre “Kola Science Centre”, 184209 Apatity, Russia; a.vilnet@ksc.ru

**Keywords:** diversity, filamentous cyanobacteria, morphology, molecular phylogeny, polyphasic approach, 16S rRNA gene, 16S–23S ITS rRNA, hypothetical secondary structures

## Abstract

During a study of cyanobacterial colonization on coal ash dumps at the Apatity Thermal Power Plant (Murmansk Region, NW Russia), two strains of *Nostoc*-like morphotypes were collected, cultivated, and examined using a polyphasic approach. Both strains KPABG–133801 and KPABG–133804 exhibited high similarity in the 16S rRNA gene (99.93%) and identical 16S–23S ITS regions. Phylogenetically, they provided robustly supported affinity to the genus *Pseudoaliinostoc*, which currently comprises eight species predominantly distributed in Southeast Asia. The similarity of the 16S rRNA gene (95.74–97.25%), the divergence in the 16S–23S ITS rRNA region (18.56–26.28%), and the unique hypothetical secondary structures of conserved helices (D1–D1′, Box–B, V3) strongly suggest that these Arctic strains represent a new species, *Pseudoaliinostoc murmanicum,* which is described and illustrated in this study. The species forms bright blue-green colonies that gradually turn olive-green with age and is characterized by elongated cells in young trichomes, vegetative cell width of less than 3.2 µm, and the presence of akinetes wider than 3.5 µm.

## 1. Introduction

The modern taxonomy of cyanobacteria is being significantly revised through a polyphasic approach, integrating morphological, ecological, and molecular data. This has resulted in the reorganization of traditional genera and the description of new monophyletic taxa. The taxonomy of the genus *Nostoc* has been revised to achieve monophyly. As a result, several new genera have been separated from *Nostoc* sensu stricto, including *Mojavia* [1], *Desmonostoc* [2], *Halotia* [3], *Aliinostoc* [4], *Komarekiella* [5], *Desikacharya* [6], *Minunostoc* [7], *Compactonostoc* [8], *Purpureonostoc* [9], *Violetonostoc* [10], *Amazonocrinis*, *Atlanticothrix*, *Dendronalium* [11], *Parakomarekiella* [12], *Pseudoaliinostoc* [13], and *Ahomia* [14]. These lineages are morphologically similar but phylogenetically distinct and distant from members of *Nostoc* sensu stricto.

Recently, a generic status was proposed for one of these phylogenetic lineages under the name *Pseudoaliinostoc* [13]. Representatives of this genus form a morphological complex that is virtually indistinguishable from typical *Aliinostoc* representatives under light microscopy, exhibiting similar filamentous thalli with oval heterocysts and large, almost round or oval akinetes. Key diagnostic differences between these genera lie in the molecular realm and include a unique number of substitutions in conserved regions of the 16S rRNA gene, as well as a high degree of divergence in the hypervariable segments of the 16S–23S ITS region.

The genus *Aliinostoc*, with the type species *Aliinostoc morphoplasticum* Bagchi, Dubey, et P. Singh from India, was established by Bagchi et al. [4]. Subsequently, *Aliinostoc soli* Saraf et al. and *Aliinostoc tiwarii* Saraf et al., also from India [15], and *Aliinostoc constrictum* Kabirnataj et al. from Iran [16] were described as new to science. During the description of a new species from the Republic of Korea, several *Aliinostoc* species were found to be distinct from the type species *A. morphoplasticum*, revealing significant divergence in the 16S rRNA gene despite their sister relationships on the phylogenetic tree. This was considered a sufficient justification for the establishment of a new genus, *Pseudoaliinostoc* [13]. In that study, the species *Pseudoaliinostoc sejongense* Bang et al. was described and three species were reclassified—*P. soli* (Saraf et al.) Lee et al. (as a type species), *P. tiwarii* (Saraf et al.) Lee et al., and *P. constrictum* (Kabirnataj et al.) Lee et al. [13]. Subsequently, two new species, *Pseudoaliinostoc yunnanense* Cai et Li and *Pseudoaliinostoc jiangxiense* Cai et Li, were proposed from China [17]. The significantly expanded dataset of *Nostoc*-like taxa in Cai et al. [17] further demonstrated the distant phylogenetic positions of the genera *Aliinostoc* and *Pseudoaliinostoc*. The most recently described species in the genus are *Pseudoaliinostoc granulatum* H. Pant et P. Singh and *Pseudoaliinostoc natans* H. Pant et P. Singh from India [18]. Currently, the genus *Pseudoaliinostoc* comprises eight species, predominantly distributed in Southeast Asian regions.

As part of an ongoing study of cyanobacterial biodiversity on technogenic substrates in the Murmansk Region of Russia, strains designated KPABG–133801 and KPABG–133804 were isolated from the ash dumps of the Apatity Thermal Power Plant [19]. Initial morphological analysis allowed their preliminary assignment to the nostocalean cyanobacterial morphocomplex. Subsequent phylogenetic analysis of the 16S rRNA sequences and a detailed examination of the secondary structure of the 16S–23S ITS region unambiguously demonstrated that these strains belong to the recently described genus *Pseudoaliinostoc*. A comparison with known species of this genus revealed significant genetic differences and morphological apomorphies. In this article, we describe the new species and discuss its phylogenetic position, unique molecular and morphological characteristics, thus contributing to the refinement of the taxonomic boundaries within this genus.

Molecular phylogenetic approaches in the study of cyanobacteria have provided exciting data on their current distribution, which extends from tropical regions to the high Arctic. Examples of cyanobacterial taxa identified far from their original description region are exceedingly numerous; here, we will cite only those cases where closely related species have been recorded in high-latitude areas. For instance, the genus *Inacoccus*, originally described by Gama et al. [20] from terrestrial habitats in the Brazilian Atlantic Forest, was recently recorded in anthropogenic soil in the European Arctic [21]. Similarly, the genus *Drouetiella*, whose type species was described in Germany [22], was later reported from the Republic of Korea [23] as well as from several localities in the Russian Arctic [24]. Similarly, *Chalicogloea*, first described in caves in Spain [25], has subsequently been recorded on Baffin Island [26] and Svalbard [27]. This further underscores the need for a more detailed investigation of Arctic ecosystems and indicates gaps in data on taxon distribution.

## 2. Results

### 2.1. Taxonomic Description

**Figure 1:**
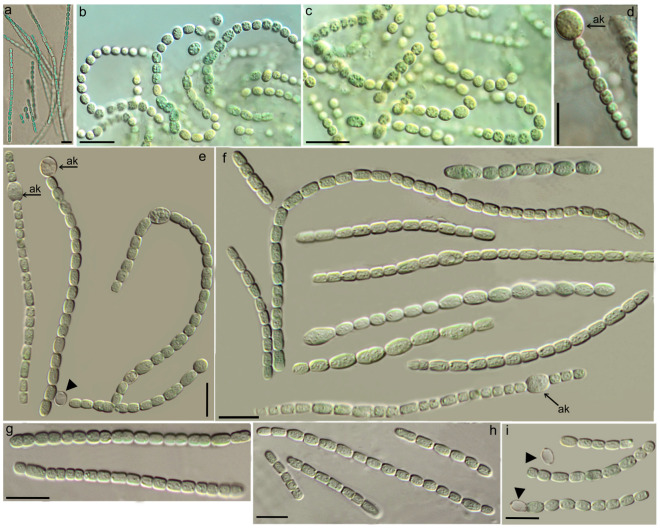
Light micrographs of the *Pseudoaliinostoc murmanicum* KPABG–133801. Scale bar = 10 μm: (**a**,**e**–**i**) filaments in a solid medium; (**b**–**d**) filaments in a liquid medium; (**e**,**f**) irregular length of cells in mature trichomes; (**d**–**f**) akinetes (ak) in both terminal and intercalary positions. Heterocytes indicated of a triangle.

*Pseudoaliinostoc murmanicum* D. Davydov & Vilnet sp. nov. (Figure 1).

Diagnosis: *Pseudoaliinostoc murmanicum* is phenotypically distinct from other species of the genus *Pseudoaliinostoc* by its long cells up to 7.4 µm in young trichomes, vegetative cells less than 3.2 µm width, and the presence of akinetes wider than 3.5 µm. It is characterized by a distinct phylogenetic position based on 16S rRNA gene and 16S–23S ITS rRNA analyses, as well as unique hypothetical secondary structures of the D1–D1′, Box–B, and V3 helices.

The similarity of the 16S rRNA gene (95.74–97.25%) did not exceed the species threshold for cyanobacteria (<98.7%) [28], while the significant dissimilarity in the 16S–23S ITS rRNA region (18.56–26.28%) exceeded the 7% level of species differentiation suggested by [29].

**Figure 2:**
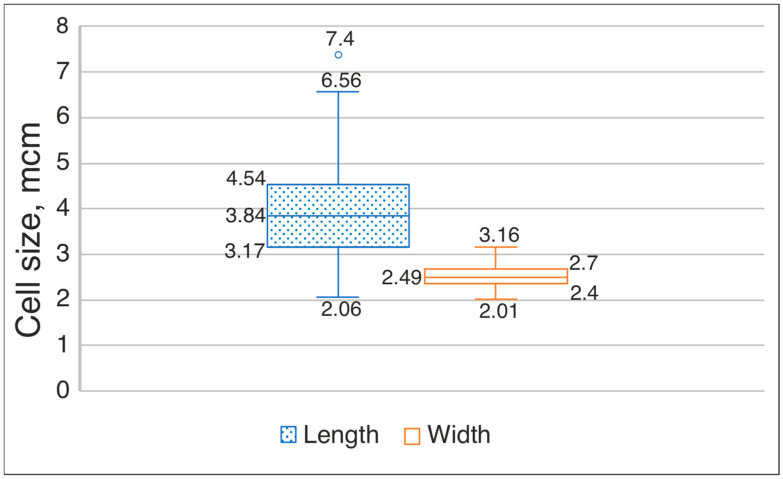
Variability in cell size of *Pseudoaliinostoc* strains KPABG–133801 and KPABG–133804.

Description: A macroscopic thallus was not observed in the field. Colonies on solid media formed bright blue-green growths on the agar surface, which gradually turned olive-green with prolonged cultivation. When cultured in a liquid medium, flocculent masses are formed, which float freely in the medium. Filaments exhibit gliding motility, are straight or curved, and less frequently coiled into loops. Filaments are isopolar and non-branching. The sheath is indistinct. Trichomes are constricted at the cross-walls. The shape of terminal cells is rounded, or attenuated, or slightly tapered. Vegetative cells appear granular; in young trichomes, vegetative cells are rectangular or square, while in matured trichomes they are usually barrel-shaped to spherical. In young trichomes, cell length is more variable than width, with a median cell length of 3.84 µm, ranging from short cells of 2.06 µm to very long cells up to 7.4 µm. The average cell length at different stages is 3.17–4.54 ± 0.99 µm, and width is 2.36–2.69 ± 0.23 µm (Figure 2). The high variability in longitudinal dimensions, expressed as a coefficient of dispersion, approaches 1 (0.994). Cell width is more stable. The median width is 2.49 µm, and the dispersion of this trait is insignificant—0.051. Heterocytes located in both terminal and intercalary positions. Heterocyte length is 4.77–7.9 µm, width is 2.92–5.03 µm; the average dimensions of heterocytes are length 5.99 µm, width 3.83 µm; median values for length are 5.99 µm, for width 3.77 µm. Akinetes are spherical or subspherical, located in both terminal and intercalary positions. The dimensions of akinetes significantly exceed those of vegetative cells. Length (5.1)5.8–7.3(8.4) µm, width (4.35)6.2–7.3(8.3) µm.

Etymology: *murmanicum* (N.L. masc. Adj.) refers to Murmansk Region where the strain was isolated, transliterated into Latin.

Type Locality: The ash dumps of the Apatity CHP, 3-year-old dump. Murmansk Region, Russia. 67.5999° N, 33.48089° E, elevation: 180 m, collected by D. Davydov on 4 June 2021.

Habitat: The crust on the ground.

Holotype: KPABG–4467 (dried cultured material, preserved in a metabolically inactive state) in the herbarium of Polar-Alpine Botanical Garden-Institute of Kola Science Centre of RAS, Kirovsk, Murmansk region, Russia.

Reference strain: KPABG–133801 in the Collection of Cyanoprokaryotes of Polar-Alpine Botanical Garden-Institute of Kola Science Centre of RAS, Kirovsk, Murmansk region, Russia.

Nucleotide sequence: GenBank (National Center for Biotechnology Information, NCBI) accession number is PV645128 for the 16S gene and 16S–23S ITS rRNA.

The paratype specimen was KPABG–4469 (dried cultured material of the strain KPABG–133804, preserved in a metabolically inactive state). The sequence of the strain KPABG–133804 was deposited in GenBank under the accession number PV645129.

### 2.2. Molecular Analyses

**Figure 3:**
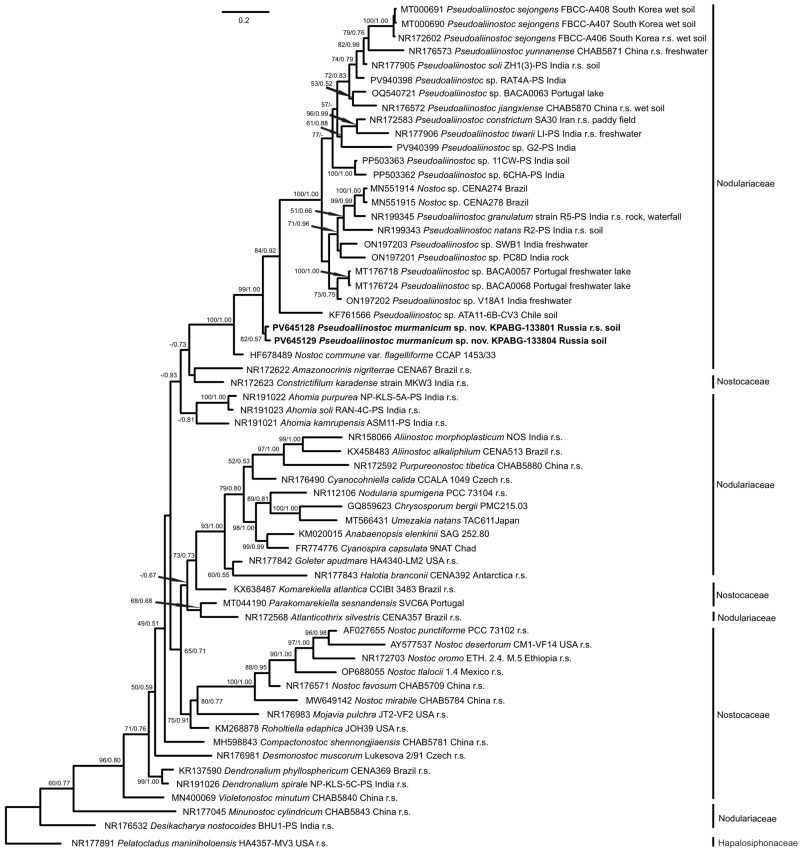
Phylogram obtained by the Bayesian approach based on 16S rRNA gene sequences from members of the families Nodulariaceae and Nostocaceae. Bootstrap support values and Bayesian posterior probabilities greater than 50% (0.50) are indicated above the nodes. Reference strains are marked as “r.s.”. GenBank accession numbers, strain designations, and geographical origins are provided. For strains of the genus *Pseudoaliinostoc*, the habitat is indicated where known.

**Figure 4:**
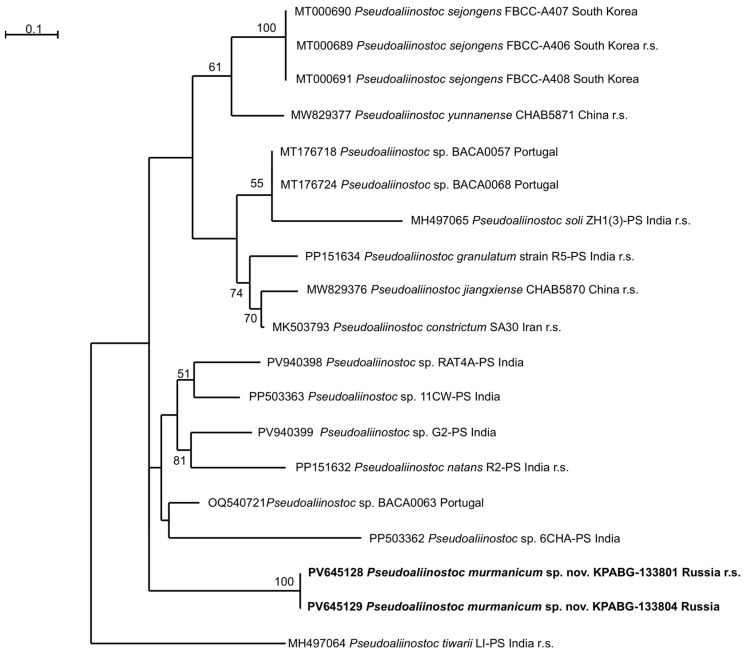
Maximum likelihood phylogram based on 16S–23S ITS region sequences for the genus *Pseudoaliinostoc*. Bootstrap support values greater than 50% are indicated. The reference strains are marked as “r.s.”.

The ML analysis of the 16S rRNA gene dataset produced a tree with a log-likelihood of −8086.960. The Bayesian analysis resulted in trees with arithmetic means of log-likelihood −8136.39 and −8136.03 for the two sampling runs. The phylogenetic trees obtained by both methods were congruent in their topologies (Figure 3). The ML analysis of the 16S–23S ITS rRNA region produced a tree with a log-likelihood of −3467.391 (Figure 4).

The strains KPABG–133801 and KPABG–133804 formed a single clade that showed a sister relationship to a clade composed of species of the genus *Pseudoaliinostoc*. This affinity received a high support from both calculations (bootstrap support (BS) 99% ML/posterior probability (PP) 1.00 BA or 99/1.00) in the 16S rRNA gene topology. The next divergence on the tree corresponded to *Nostoc commune* var. *flagelliforme* strain CCAP 1453/33 (HF678489), which may be misidentified. The genera of both families sampled, Nostocaceae and Nodulariaceae, were intermingled on trees and formed mixed clades. The internal nodes of the tree were poorly supported, which did not allow robust clarification of phylogenetic affinities among *Nostoc*-like genera. The genus *Pseudoaliinostoc* revealed an unsupported relationship with the genera *Amazonocrinis* and *Constrictifilum* of Nostocaceae. The selection of taxa for phylogenetic estimation affected topology and the relationships of genera, which appear to be slightly different in subsequent studies [14,17,18].

The 16S–23S ITS rRNA topology (Figure 4) had not obtained supports for the internal nodes, so the relationship between species remained unclear. Both tested strains, KPABG–133801 and KPABG–133804, were placed in a distinct subclade without close affinity to any known species of *Pseudoaliinostoc* or previously sequenced strains.

The similarity of the 16S rRNA gene between strains KPABG–133801 and KPABG–133804 was 99.93% (Table 1). Their similarity to reference strains of eight known *Pseudoaliinostoc* species ranged from 95.74% (with *P. yunnanense* CHAB5871) to 97.25% (with *P. granulatum* R5-PS). The 16S–23S ITS rRNA region was identical in both tested strains, and its dissimilarity to that of other species ranged from 18.55% to 26.28%.

The reference strains *Pseudoaliinostoc soli* ZH1(3)-PS, *P. tiwarii* LI-PS, and *P. constrictum* SA30 were characterized by short 16S–23S ITS rRNA region fragments that lack the tRNA^Ile^ and tRNA^Ala^ genes. Therefore, only the length and hypothetical secondary structures of the D1–D1′ helix could be compared across all known *Pseudoaliinostoc* species. The longest D1–D1′ helix, comprising 66 nucleotides (bp), was recorded for *Pseudoaliinostoc soli* ZH1(3)-PS, while the shortest, with 62 nucleotides, was found in *P. constrictum* SA30 (Table 2). The other six species and the tested strains possessed a D1–D1′ helix length of 65 nucleotides. The *Pseudoaliinostoc natans* R2-PS proved a robust secondary D1–D1′ helix structure for length of 54 nucleotides, while D1–D1′ alignment for it could count 65 nucleotides as well.

The length of the Box–B helix was more variable: 26 nucleotides in *Pseudoaliinostoc granulatum* R5-PS and 33 nucleotides in *P. jiangxiense* CHAB5870. The strains *P. natans* R2-PS, *P. sejongens* FBCC-A406, and *P. yunnanense* CHAB5871 had a Box–B length of 29 nucleotides. The tested strains from the Murmansk Region exhibited a unique Box–B length of 30 nucleotides.

The V3 helix showed an intermediate level of length variability: the shortest length, 35 nucleotides, was found in *P. natans* R2-PS, while the longest, 38 nucleotides, occurred in the tested strains KPABG–133801 and KPABG–133804. The remaining strains had a V3 helix length of 36 nucleotides.

**Figure 5:**
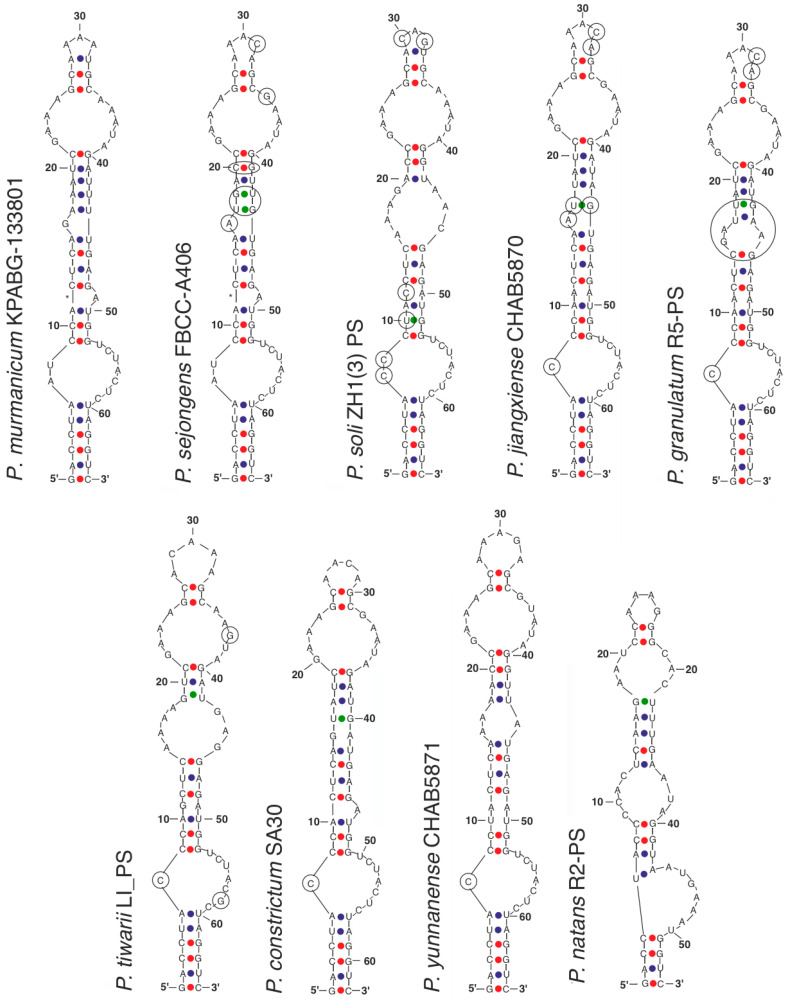
The secondary structure of D1–D1′ helices of the 16S–23S ITS rRNA region of *Pseudoaliinostoc* strains. Circles indicate differences.

The helices D1–D1′ of the most species had an identical basal stem of six nucleotide pairs, except for *Pseudoaliinostoc yunnanense* CHAB5871, which had five pairs, and *P. natans*, which had four (Figure 5). Following the basal stem, a basal loop is formed, which was similar in nucleotide composition across species. Most species had cytosine at the seventh position within this basal loop (*Pseudoaliinostoc granulatum*, *P. jiangxiense*, *P. tiwarii*, *P. constrictum*). Exceptions were *P. sejongens*, *P. murmanicum* (A-U), and *P. soli* (C-C).

The structure of the intermediate stem in *Pseudoaliinostoc murmanicum* was the most similar to that of *Pseudoaliinostoc sejongens*. Differences began at position 16, where guanine was present instead of adenine, as well as in the next two pairs (A-U, A-U instead of U-G, G-U) and U-A at position 20 instead of C-G. At position 36, the guanine found in *P. sejongens* was replaced by adenine.

**Figure 6:**
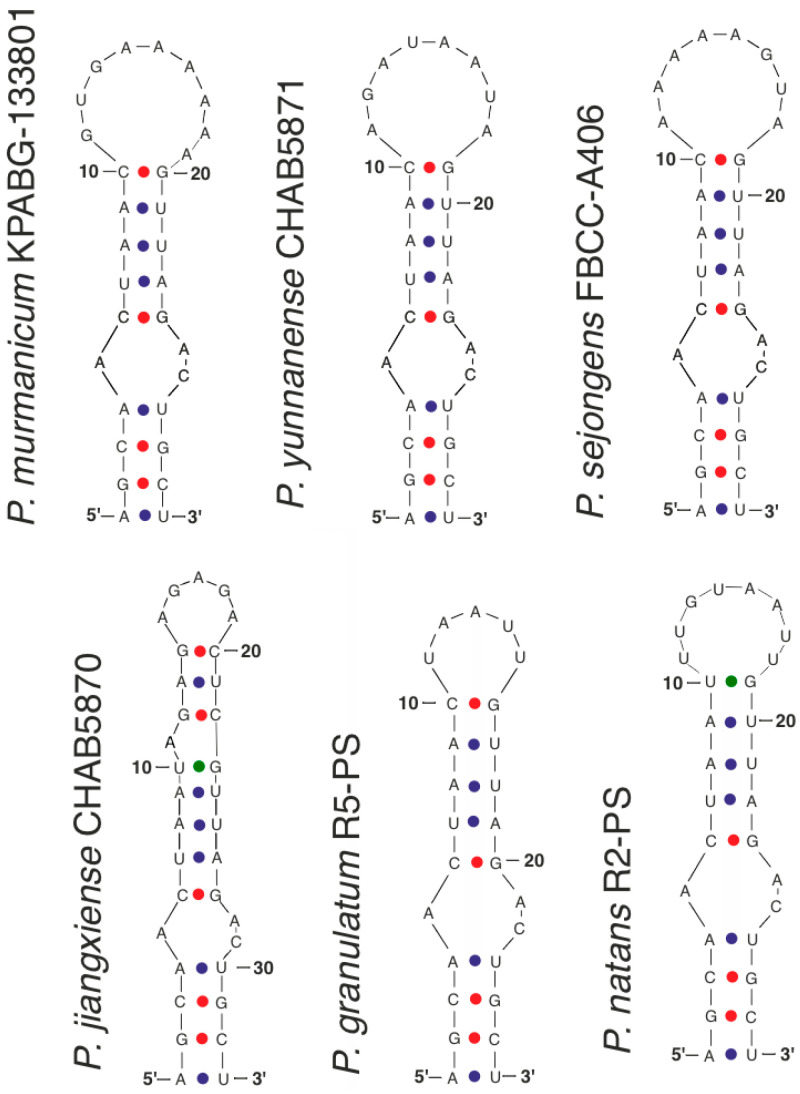
The secondary structure of Box–B helices of the 16S–23S ITS rRNA region of *Pseudoaliinostoc* strains.

The Box–B helix structure was highly conserved among most of the taxa compared. Observed differences resided primarily within the variable terminal loop (Figure 6). In *Pseudoaliinostoc murmanicum*, the Box–B structure was similar to that of *P. sejongens* and *P. yunnanense*, differing in a larger nucleotide count and a distinct nucleotide composition within its terminal loop.

Compared to the aforementioned taxa, the Box–B helix of *Pseudoaliinostoc natans* also varied in the terminal loop structure: it contained eight nucleotides, matching the count in *P. sejongens*/*P. yunnanense*, but with a different nucleotide sequence, and additionally featured a cytosine-to-uracil substitution at the 10th position.

In *Pseudoaliinostoc granulatum*, the terminal loop comprised only five nucleotides, while the remainder of the helix structure aligned with the other species. *Pseudoaliinostoc jiangxiense* exhibited a basal stem structure consistent with the general model of the genus; however, significant deviations began after the 10th nucleotide pair. More substantial differences were observed in *Pseudoaliinostoc constrictum*, which was characterized by the formation of an extended subbasal loop containing a greater number of nucleotides.

**Figure 7:**
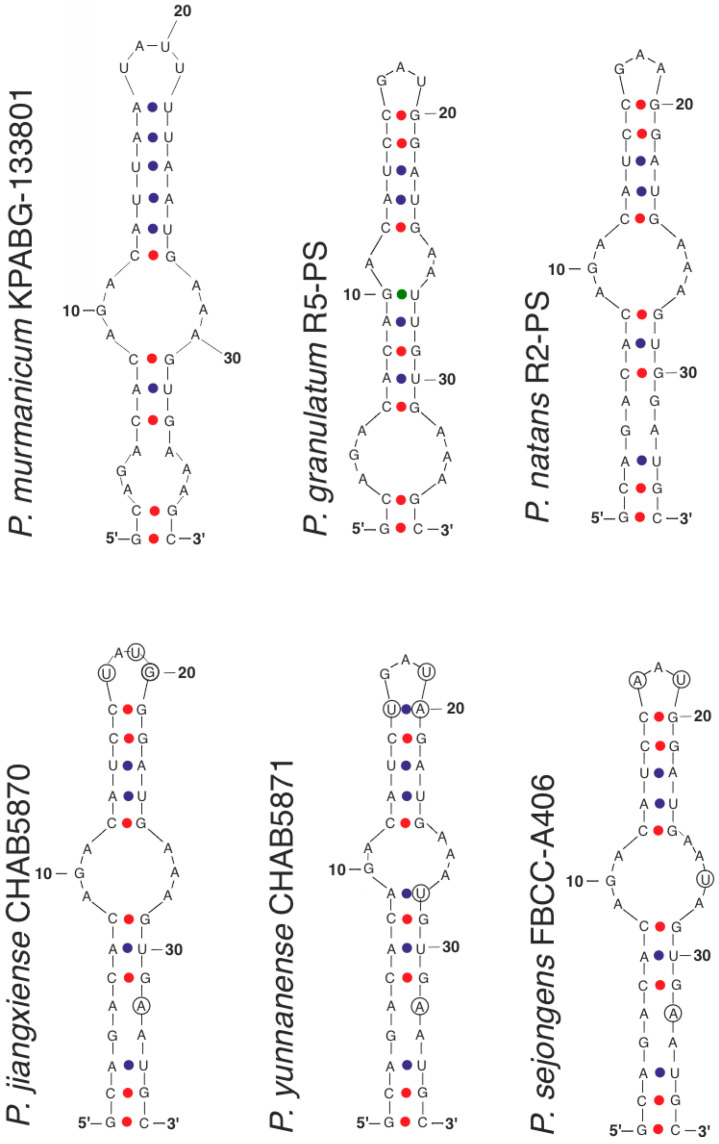
The secondary structure of V3 helices of the 16S–23S ITS rRNA region of *Pseudoaliinostoc* strains. Circles indicate differences.

The V3 helix structure in *Pseudoaliinostoc murmanicum* differed significantly from the common type characteristic of *Pseudoaliinostoc jiangxiense*, *P. natans*, *P. sejongens*, and *P. yunnanense* (Figure 7). In addition to the median and apical loops, *Pseudoaliinostoc murmanicum* also exhibited a basal loop, which was partly similar in its nucleotide composition to that of *P. granulatum*. Furthermore, instead of the C-G, C-G base pairs in the subapical position typical of most species (*Pseudoaliinostoc jiangxiense*, *P. granulatum*, *P. natans*, *P. sejongens*), *Pseudoaliinostoc murmanicum* displayed A-U, A-U pairs.

Differences in the V3 helix structure among the strains *Pseudoaliinostoc jiangxiense*, *P. natans*, *P. sejongens*, and *P. yunnanense* were minor and involved substitutions or insertions of individual nucleotides (Figure 7, indicated by circles).

## 3. Discussion

Nostocoid cyanobacteria possess a limited set of morphological characteristics. Delimitation of individual monophyletic lineages at the genus level often lacks reliable and obvious autapomorphies. Unbranched, uniseriate, and isopolar filamentous cyanobacteria with either intercalary or basal heterocytes and the frequent occurrence of arthrospores constitute the family Nodulariaceae [30]. Reliable delimitation of taxa within this family is achievable only through molecular genetic data. Three species of *Aliinostoc* were transferred to a separate genus, *Pseudoaliinostoc*, based on significant genetic distances [13]. In recent years, five more species have been described [13,17,18] and remote phylogenetic affinity of both genera was proved [17,18]. But, morphologically, representatives of *Pseudoaliinostoc* form a complex that is practically indistinguishable from typical representatives of *Aliinostoc*, exhibiting similar filamentous thalli with oval heterocytes and large, almost round or oval akinetes.

This research applied a polyphasic approach to characterize two strains, KPABG–133801 and KPABG–133804, isolated from the ash dump in the European part of the Russian Arctic. Preliminary morphological analysis placed them within the *Nostoc*-like cyanobacterial group. On the phylogenetic tree of the 16S rRNA gene, strains KPABG–133801 and KPABG–133804 formed a separate subclade within the genus *Pseudoaliinostoc* species. Both strains shared 99.93% similarity in the 16S rRNA gene and identity in the 16S–23S ITS region. These values reveal consistent differences from all currently described *Pseudoaliinostoc* species in both molecular markers, meeting the established thresholds for species delimitation in Cyanobacteriota. The hypothetical secondary structures of the conserved D1–D1′, Box–B, and V3 helices are unique and provide further support for the recognition of the Arctic strains as a distinct species within the genus.

A comparison of the morphology of established *Pseudoaliinostoc* species reveals that they all share the characteristics of uniseriate, isopolar filaments, trichomes with distinct constrictions, and intercalary/terminal heterocytes (Table 3). Although the morphological features of these species often fall within overlapping ranges, the combination of trait sets allows their morphological identification, which is reflected in the species identification key.

*Pseudoaliinostoc murmanicum* differs from three species (*P. constrictum*, *P. granulatum*, *P. natans*) due to the presence of akinetes. It differs from *Pseudoaliinostoc jiangxiense* by having akinetes wider than 3.5 µm. A vegetative cell width of less than 3.2 µm distinguishes *Pseudoaliinostoc murmanicum* from the species *P. sejongens*, *P. soli*, *P. tiwarii*, and *P. yunnanense*.

### 3.1. Key to Identified Species of Pseudoaliinostoc, Based on Morphology

1a. Akinetes absent21b. Akinetes present42a. Heterocytes width predominantly up to 3.0 µm
*P. natans*
2b. Heterocytes width predominantly more than 3.0 µm33a. Vegetative cell width 2.2–3.2 µm
*P. granulatum*
3b. Vegetative cell width 3.1–5.6 µm
*P. constrictum*
4a. Akinete width up to 3.5 µm
*P. jiangxiense*
4b. Akinete width more than 3.5 µm55a. Vegetative cell width predominantly more than 4.5 µm65b. Vegetative cell width predominantly less than 4.5 µm76a. Vegetative cell width less than 5.2 µm
*P. sejongens*
6b. Vegetative cell width more than 5.2 µm
*P. tiwarii*
7a. Vegetative cell width less than 3.2 µm
*P. murmanicum*
7b. Vegetative cell width more than 3.2 µm88a. Akinete width less than 4.5 µm
*P. yunnanense*
8b. Akinete width more than 4.5 µm
*P. soli*


### 3.2. Ecology of Pseudoaliinostoc Species

Most strains assigned to *Pseudoaliinostoc* have been isolated from soil samples (*P. jiangxiense*, *P. natans*, *P. sejongens*, *P. soli*) [13,15,17,18]. The strains *Pseudoaliinostoc* sp. ATA11-6B-CV3 (KF761566) and *Pseudoaliinostoc* sp. 11CW-PS (PP503363) were isolated from soil in Chile and India, respectively. A similar habitat type is likely represented by ash dumps, where colonies of *P. murmanicum* were discovered. The strain *Pseudoaliinostoc* sp. (PC8D, ON197201) was found on rocks in India. *Pseudoaliinostoc constrictum* (found in a paddy field) [16] and *Pseudoaliinostoc granulatum* (found on rock under a waterfall) [18] inhabit transitional sub-aerophytic environments.

*Pseudoaliinostoc tiwarii* [15], and *P. yunnanense* [17] are inhabitants of freshwater habitats. Unpublished records suggest that *Pseudoaliinostoc sejongens* (OQ291372) is capable of occupying similar freshwater habitats in India, along with the unidentified strains *Pseudoaliinostoc* sp. SWB1 (ON197203) and *Pseudoaliinostoc* sp. V18A1 (ON197202). In Southern Europe, strains of *Pseudoaliinostoc* sp. BACA0063 (OQ540721), BACA0057 (MT176718), and BACA0068 (MT176724) have also been recorded in lake waters.

### 3.3. Distribution of Pseudoaliinostoc Species

**Figure 8:**
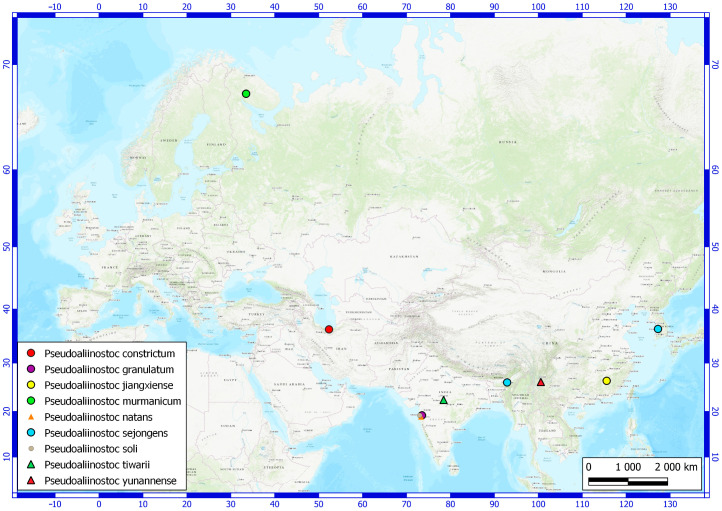
Distribution of molecularly confirmed *Pseudoaliinostoc* species across various geographic regions.

Currently, data on the distribution of *Pseudoaliinostoc* species remain quite limited. Most species are associated with tropical and subtropical climates—they have been found in Southeast Asia (Figure 8). The exceptions are the finding of *Pseudoaliinostoc constrictum* in Iran [16], and in the northernmost location, the discovery of *P. murmanicum*.

Regarding strains recorded in the NCBI database that have not been identified as any known *Pseudoaliinostoc* species, all occurrences are also confined to the subtropical zone—India, the Azores [31], or South America (Atacama, Chile, KF761566).

The discovery of *P. murmanicum* in the European Arctic of Russia represents the northernmost record of the genus *Pseudoaliinostoc* worldwide.

## 4. Materials and Methods

### 4.1. Description of Study Sites

The study was conducted at the ash disposal site of the Apatity Combined Heat and Power (CHP) Plant, commissioned in 1959. The site is located in the valley of the Belaya River, on a plain within the Khibiny Mountains foothills in the central Murmansk Region (northern taiga subzone). The combustion of pulverized coal at the plant generates ash and slag, which are hydraulically transported with process water to the disposal area. Cyanobacterial strains KPABG–133801 (67.5999° N, 33.48089° E) and KPABG–133804 (67.599972° N, 33.48128° E) were collected from the surface layer (0–3 cm depth) of a three-year-old ash dump using 10 cm × 10 cm quadrats [19].

### 4.2. Culture Condition and Morphological Study

Soil suspensions from each sample were used to inoculate both liquid and agarized Z8 nutrient medium [32]. The cultures were maintained at 22 °C under a 16:8 h light:dark photoperiod, with an illumination of 35 μmol photons m^−2^ s^−1^. Monospecies cyanobacterial cultures were maintained by serial passages between solid and liquid media.

For morphological analysis, we examined three-week-old cultures using an AxioScope A1 microscope (Zeiss AG, Jena, Germany) equipped with Nomarski interference contrast and an Olympus DP23 camera (Tokyo, Japan). Morphometric data was acquired from 100 measurements per isolate characteristic using an Olympus cellSens Entry 3.2 system (Tokyo, Japan), with measurements taken from various positions on the slide. Measurements were taken from multiple culture replicates and from different filaments at various growth stages to ensure representative sampling.

### 4.3. DNA Extraction and Sequencing

The cyanobacterial cells were harvested from the cultures KPABG–133801 and KPABG–133804, and the total genomic DNA of the strain were extracted using the DNeasy Plant Mini Kit (Qiagen, Venlo, Limburg, The Netherlands) following the manufacturer’s instructions.

The primers for the amplification of the partial 16S rRNA gene and the 16S–23S ITS rRNA region, as suggested by Neilan et al. [33] (27F: 5′–AGA GTT TGA TCC TGG CTC AG–3′) and Boyer et al. [34] (1: 5′–CTC TGT GTG CCT AGG TAT CC–3′), were used. The PCR was performed in 20 µL reaction volumes containing MasDDTaqMIX (Dialat Ltd., Moscow, Russia), 10 pmol of each oligonucleotide primer, and 1 ng of DNA with the following amplification cycles: 3 min at 94 °C, 40 cycles (30 s 94 °C, 40 s 56 °C, 60 s 72 °C) and a final extension at 72 °C for 2 min. The amplicons were visualized on 1% agarose TAE gels by EthBr staining, purified using the Cleanup Mini Kit (Evrogen, Moscow, Russia), and then used as templates for sequencing reactions with the ABI Prism BigDye Terminator v. 3.1 Kit (Applied Biosystems, Waltham, MA, USA), following the standard protocol provided for 3730 Avant Genetic Analyzer (Applied Biosystems, Waltham, MA, USA). Additionally, the internal primer 2 (5′–GGG GGA TTT TCC GCA ATG GG–3′) from [35] was used for sequencing. A single operon was obtained for each strain, with no ambiguities across the entire 16S–23S rRNA region, which contained both tRNA genes (tRNA^Ile^ and tRNA^Ala^).

### 4.4. Molecular Phylogenetic Analyses

The nucleotide sequence of the 16S–23S rRNA region for two tested strains was assembled using BioEdit 7.0.1 [36]. Preliminary identification of both strains was carried out using the BLAST algorithm (https://blast.ncbi.nlm.nih.gov). The number of the reference strains of the genus *Pseudoaliinostoc* was found as approximately 98% similar from the 16S rRNA gene. To produce an appropriate dataset for determining the phylogenetic position of the tested strains, the modern classification schemes of [30,37] were used. Accessions from reference strains of *Nostoc*-like species belonging to the families Nodulariaceae Elenkin and Nostocaceae Agardh ex Kirchner were downloaded from GenBank. The family Nodulariaceae was represented by 16 genera and 26 species, while the family Nostocaceae was represented by 10 genera and 17 species. Additionally, 14 strains related to the eight described species of the genus *Pseudoaliinostoc* were included.

*Godleya alpina* Novis et Visnovsky, from the related family Tolypotrichaceae, was chosen as an outgroup. The 16S rRNA gene dataset comprises 61 accessions and has a total length of 1422 sites. The 16S–23S ITS rRNA region contains sequences from 19 accessions, including eight reference strains and two tested strains, and comprises 598 sites.

The maximum likelihood (ML) method using W-IQ-TREE [38] and the Bayesian approach with MrBayes v3.2.1 [39] were used to reconstruct the molecular phylogeny based on the 16S rRNA gene. The ML analysis was performed using the best-fit evolutionary model of nucleotide substitutions and ultrafast bootstrapping [40]. ModelFinder [41] identified GTR+F+R3 as the best model for the 16S rRNA gene. For Bayesian analysis (BA), the GTR+I+G model was applied. Two independent runs of the Metropolis-coupled MCMC were performed to sample parameter values according to their posterior probability. Each run included three heated chains and one unheated chain, with two starting trees chosen randomly. The chains were run for five million generations, and trees were sampled every 100th generation. The first 12,500 trees from each run were discarded as burn-in, resulting in 75,000 sampled trees. The average standard deviation of the split frequencies between the two runs was 0.007423. Bayesian posterior probabilities were calculated from trees sampled after the burn-in procedure. Majority rule consensus trees were obtained after combining the runs, excluding the 25% burn-in.

The phylogenetic relationship within the genus *Pseudoaliinostoc* based on the 16S–23S ITS rRNA region was estimated using the ML method with the GTR+F+G4 nucleotide substitution model and ultrafast bootstrapping.

The nucleotide sequence similarity of the 16S rRNA gene was estimated using the formula: S = 100 × (1 − p), where *p* represents the average pairwise *p*–distances. These distances were calculated using MEGA 11 [42] with the pairwise deletion option to account for gaps. The dissimilarity of the 16S–23S ITS region was also determined as the average pairwise *p*–distances.

To identify the conserved helices D1–D1′, Box–B and V3 in the 16S–23S ITS region, the model of 16S–23S ITS rRNA region proposed by [43] was used. Hypothetical secondary structures of all conserved helices were obtained using Mfold [44].

## 5. Conclusions

Active exploration of Cyanobacteriota diversity in the Russian Arctic using a polyphasic approach has shed light on the current distribution of both traditionally known and recently described taxa, and has led to the discovery of new species belonging to genera with predominantly more southern distributions. *Pseudoaliinostoc murmanicum* is one such recently described species, recorded from anthropogenic landscapes of the Murmansk Region, together with *Apatinema mutabile* D. Davydov [45] and *Inacoccus terrestris* D. Davydov et Vilnet [21], as well as from natural landscapes of the Russian Arctic, including *Drouetiella ramose* D. Davydov et al. [46] and *D. elegans* D. Davydov et Vilnet [47]. It highlights the adaptability of these cyanobacteria and underscores the importance of exploring extreme and under-investigated environments, such as Arctic technogenic landscapes, as reservoirs of novel microbial biodiversity.

This study contributes to the ongoing revision of nostocalean taxonomy and highlights the value of integrating molecular, morphological, and ecological data in uncovering hidden diversity, even in anthropogenically influenced habitats such as ash disposal sites.

The study of nostocalean cyanobacteria in industrial dumps and contaminated substrates is particularly valuable, as the search for and assessment of the potential of native cyanobacterial strains from Arctic habitats will enable future trials for the reclamation of technogenic substrates and contaminated soils in the Murmansk Region. The processing of mineral resources exerts a significant environmental impact through the creation of dumps and ore-dressing wastes. Such substrates directly affect the environment, as their vegetation-free and unconsolidated surfaces are subject to intense wind and water erosion, thereby polluting water, air, while also worsening the sanitary conditions of nearby towns. Due to their unique combination of photosynthesis and molecular nitrogen fixation, nostocalean cyanobacteria are among the most widespread and important primary producers in the formation of pioneer communities that colonize bare substrates. The formation of an extracellular matrix by cyanobacteria contributes to the development of biofilms and the stabilization of surfaces. Thus, there are prospects for the application of cyanobacterial communities in developing technologies for the reclamation of technogenic substrates and barren lands.

## Figures and Tables

**Table 1:** Pairwise nucleotide similarity/dissimilarity (%) within the genus *Pseudoaliinostoc* based on the 16S rRNA gene (1422 bp) and the 16S–23S ITS region (598 bp).

Taxon	Interspecific Nucleotide Sequence Similarity 16S/Dissimilarity 16–23S ITS, %								
	1	2	3	4	5	6	7	8	9
1. *P. murmanicum* KPABG–133801									
2. *P. murmanicum* KPABG–133804	99.93								
	0								
3. *P. sejongens* FBCC-A406	95.96	96.03							
	19.49	19.49							
4. *P. yunnanense* CHAB5871	95.74	95.81	97.86						
	18.56	18.56	11.55						
5. *P. soli* ZH1(3)-PS	96.69	96.77	98.79	98.58					
	-	-	-	-					
6. *P. jiangxiense* CHAB5870	96.77	96.84	98.08	97.44	98.58				
	21.06	21.06	-	19.39	17.49				
7. *P. constrictum* SA30	96.72	96.80	98.38	97.42	98.23	98.08			
	-	-	-	-	-	-			
8. *P. tiwarii* LI-PS	96.33	96.40	98.15	96.58	97.29	98.08	98.52		
	-	-	-	-	-	-	-		
9. *P. granulatum* R5-PS	97.25	97.33	97.85	97.76	98.62	98.28	97.84	97.42	
	22.75	22.55	18.69	16.00	-	12.02	-	-	
10. *P. natans* R2-PS	97.07	97.16	97.50	97.42	97.93	96.99	97.39	97.33	98.19
	26.28	26.09	26.29	24.51	-	24.71	-	-	22.37

**Table 2:** Sequence length variability (base pairs, bp) within 16S–23S ITS rRNA among reference strains of *Pseudoaliinostoc*.

Strains	D1–D1′ Helix	Box–B	V3
*P. murmanicum* KPABG–133801	65	30	38
*P. constrictum* SA30	62	-	-
*P. granulatum* R5-PS	65	26	36
*P. jiangxiense* CHAB5870	65	33	36
*P. natans* R2-PS	54 (65?)	29	35
*P. sejongens* FBCC-A406	65	29	36
*P. soli* ZH1(3)-PS	66	-	-
*P. tiwarii* LI-PS	65	-	-
*P. yunnanense* CHAB5871	65	29	36

“?”—the *Pseudoaliinostoc natans* R2-PS has secondary structure for length of 54 nucleotides, while alignment for it counts 65 nucleotides.

**Table 3:** Comparative morphological traits of *Pseudoaliinostoc* species (µm).

Traits	*P. murmanicum*	*P. constrictum*	*P. granulatum*	*P. jiangxiense*	*P. natans*	*P. sejongens*	*P. soli*	*P. tiwarii*	*P. yunnanense*
Vegetative cells length	(2.06)3.17–4.54(7.4)	2.5–6.5	1.6–2.8	3.20–4.95	2.1–2.4	2.9–5.3	3.6–4.0	5.3–5.6	2.15–5.21
Vegetative cells width	(2.01)2.36–2.69(3.16)	3.1–5.6	2.2–3.2	3.40–4.78	2.1–2.7	4.5–5.2	3.2–3.7	5.2–5.6	2.05–4.89
Heterocytes length	4.77–7.9	6.5–8.0	2.9–4.7	3.53–4.77	1.9–2.9	6.0–9.3	3.8–4.3	3.8–4.3	3.30–4.25
Heterocytes width	2.92–5.03	3.0–6.8	3.2–4.7	3.20–4.15	1.6–3.2	5.5–8.7	3.7–4.9	3.7–4.9	2.90–4.40
Akinetes length	(5.1)5.8–7.3(8.4)	absent	absent	5.01–6.72	absent	6.8–7.7	?8	?	5.19–6.00
Akinetes width	(4.35)6.2–7.3(8.3)	absent	absent	2.24–3.50	absent	7.2–8.3	?5.5	?	3.68–4.18

The “?” indicates that akinetes size data were not provided in the original species descriptions.

## Data Availability

The data supporting the reported results can be found in the publicly archived dataset of the “L” Information system https://isling.org/ (accessed on 10 March 2026).
